# Thermostabilisation of the Serotonin Transporter in a Cocaine-Bound Conformation

**DOI:** 10.1016/j.jmb.2013.03.025

**Published:** 2013-06-26

**Authors:** Saba Abdul-Hussein, Juni Andréll, Christopher G. Tate

**Affiliations:** MRC Laboratory of Molecular Biology, Hills Road, Cambridge CB2 0QH, UK

**Keywords:** GPCR, G-protein-coupled receptor, T4L, T4 lysozyme, NSS, neurotransmitter sodium symporter, DDM, *n*-dodecyl-β-d-maltopyranoside, membrane protein, thermostability, structure, neurotransmitter transporter, thermostabilisation

## Abstract

Structure determination of mammalian integral membrane proteins is challenging due to their instability upon detergent solubilisation and purification. Recent successes in the structure determination of G-protein-coupled receptors (GPCRs) resulted from the development of GPCR-specific protein engineering strategies. One of these, conformational thermostabilisation, could in theory facilitate structure determination of other membrane proteins by improving their tolerance to detergents and locking them in a specific conformation. We have therefore used this approach on the cocaine-sensitive rat serotonin transporter (SERT). Out of a panel of 554 point mutants throughout SERT, 10 were found to improve its thermostability. The most stabilising mutations were combined to make the thermostabilised mutants SAH6 (L99A + G278A + A505L) and SAH7 (L405A + P499A + A505L) that were more stable than SERT by 18 °C and 16 °C, respectively. Inhibitor binding assays showed that both of the thermostabilised SERT mutants bound [^125^I]RTI55 (β-CIT) with affinity similar to that of the wild-type transporter, although cocaine bound with increased affinity (17- to 56-fold) whilst ibogaine, imipramine and paroxetine all bound with lower affinity (up to 90-fold). Neither SAH6 nor SAH7 was capable of transporting [^3^H]serotonin into HEK293 cell lines stably expressing the mutants, although serotonin bound to them with an apparent *K*_i_ of 155 μM or 82 μM, respectively. These data combined suggest that SAH6 and SAH7 are thermostabilised in a specific cocaine-bound conformation, making them promising candidates for crystallisation. Conformational thermostabilisation is thus equally applicable to membrane proteins that are transporters in addition to those that are GPCRs.

## Graphical Abstract

**Figure:**
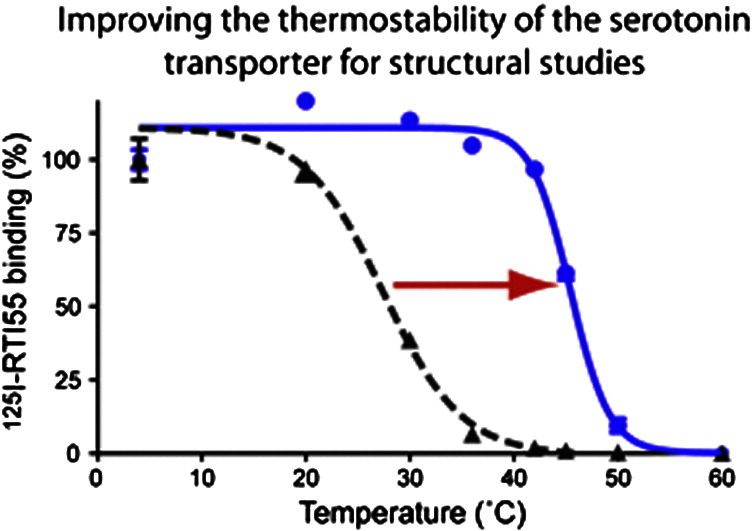

**Highlights:**

► The serotonin transporter was thermostabilised to facilitate structural studies. ► Mutant SERT-SAH6 contained three point mutations that improved thermostability up to 18 °C. ► SERT-SAH6 preferentially bound cocaine compared to other inhibitors. ► SERT-SAH6 bound serotonin with high affinity but could not transport it. ► SERT-SAH6 is locked in a cocaine-bound conformation ideal for crystallisation.

## Introduction

Structure determination of eukaryotic integral membrane proteins is challenging, but once the fundamental problems of producing well-diffracting crystals for G-protein-coupled receptors (GPCRs) had been understood and solved,^1,2^ there was a dramatic increase in the number of structures determined. A combination of strategies developed in a number of laboratories has underpinned GPCR crystallisation, including the development of receptor–T4 lysozyme (T4L) fusions,^3,4^ conformational thermostabilisation of the GPCRs,^5–9^ crystallisation in lipidic cubic phase^10,11^ and the use of antibody fragments.^12–14^ However, the key component for success is undoubtedly the stability of the GPCR during purification and crystallisation.^1,15^ Two strategies have been used successfully to improve the stability of GPCRs. The addition of high-affinity ligands to receptor–T4L fusions allowed their crystallisation in lipidic cubic phase (see, e.g., Ref. 16), with the stability of the receptor–T4L–ligand complex being determined by a fluorescent thermostability assay performed on the purified protein.^17^ An alternative strategy is to thermostabilise the receptor by systematic mutagenesis coupled to a thermostability assay based upon the binding of a radiolabelled ligand. The resulting receptor usually contains four to six thermostabilising mutations and is preferentially in a single conformation.^5,6,8,9^ The advantage of the latter strategy is that, because the receptor is thermostabilised in a specific conformation, crystal structures can be determined of the receptor bound to ligands that bind only very weakly.^18,19^ When both approaches have been used on identical or similar receptors in the same conformation, the structures of the transmembrane regions are essentially identical (rmsd ~ 0.6 Å), although larger differences may be observed in the loop regions due to lattice contacts and/or the presence of T4L.^1^

The success of developing generic strategies for the crystallisation of GPCRs suggests that analogous developments for transporters may result in a similar acceleration in the rate of their structure determination. Current methodologies for the crystallisation of transporters have relied on the identification of those transporters that are sufficiently stable for purification and crystallisation.^20^ This has allowed the structure determination of many transporters from different families, but the majority of the structures are of bacterial proteins.^21^ Clearly, purified transporters have to be sufficiently stable in detergent solution for structure determination; thus, application of thermostabilisation strategies to transporters should be possible and would facilitate structure determination. To initiate this approach for transporters, we have therefore decided to apply the strategy of conformational thermostabilisation to the rat serotonin transporter (SERT).^22^

SERT is a member of the SLC6 subclass of the neurotransmitter sodium symporter (NSS) family.^23^ Members of the NSS family play important roles in regulating neurotransmitter concentrations in the peripheral and central nervous system by re-uptake into the presynaptic nerve termini. Indeed, the monoamine transporters are key targets for therapeutic intervention in a wide range of CNS disorders, as well as the primary targets for drugs of abuse such as cocaine and amphetamines.^24,25^ SLC6 transporters in mammals are characterised by 12 transmembrane helices with a large extracellular loop between transmembrane helices 3 and 4 (TM3 and TM4) that is invariably N-glycosylated. Structural studies on this family of transporters have focused on bacterial homologues that are extremely stable, such as LeuT,^26^ but to fully understand inhibitor binding and the mechanism of transport of the mammalian transporters, it is essential to determine their structures. However, the mammalian transporters are difficult targets for structural studies. Heterologous expression of the transporters for 5HT (SERT), GABA (GAT) and norepinephrine (NET) using the baculovirus expression is possible,^27^ but functional expression levels are low and only a proportion of the expressed protein is correctly folded (as defined by the binding of radiolabelled inhibitors).^28,29^ It appears that the folding of these transporters is relatively inefficient and requires the transporter to be N-glycosylated,^28^ which allows interaction with the molecular chaperone calnexin.^29^ A parallel study of seven different expression systems clearly identified a tetracycline-inducible mammalian cell line as the most efficient at producing fully functional SERT.^30^ However, it was found that SERT is unstable in detergent solution, perhaps due to its absolute requirement for cholesterol.^31^ The advantage of working with SERT is that there is a very high affinity radiolabelled compound available, [^125^I]RTI55 (β-CIT),^28,32^ that allows the discrimination of functional *versus* misfolded SERT. We have therefore chosen SERT as an ideal, although difficult, target for thermostabilisation. Here we describe the thermostabilisation strategy and the characterisation of two thermostable mutants suitable for structural studies.

## Results

### Development of a thermostability assay for SERT

Conformational thermostabilisation of a membrane protein requires the testing of hundreds of mutants for thermostability, and therefore, a rapid and simple expression system for SERT was needed to provide sufficient transporter for the assays. The neuronal cell line HEK293 was previously identified as the most efficient system for the production of SERT^30^; thus, transient transfection was tested using a plasmid (pcDNA3) that constitutively expressed the fluorescent fusion protein SERT-mCherry from the strong cytomegalovirus promoter. However, despite altering the amount of plasmid transfected between 0.1 and 1.0 μg per 50,000 cells, the vast majority of the fusion protein appeared to be expressed intracellularly rather than at the cell surface (Abdul-Hussein, unpublished data). Previous work suggests that the intracellular material is misfolded and cannot bind radiolabelled inhibitor.^30,33^ Similar trials using transient transfection of SERT-mCherry expressed from a tetracycline-inducible promoter pcDNA5/FRT/TO identified 0.05–0.1 μg of plasmid per 50,000 cells as the optimal amount to ensure that the majority of SERT was expressed at the cell surface as observed by confocal microscopy (Fig. S1). Binding assays using an excess of [^125^I]RTI55 at a concentration of 1 nM (5 times the *K*_D_)^28^ showed that there were, on average, approximately 100,000 copies per transfected cell and that there were sufficient molecules of SERT in 50,000 cells per well of a 96-well plate to perform a single-point thermostability assay in duplicate (Fig. S2).

Thermostability assays usually involved adding the radioligand to detergent-solubilised membrane proteins,^9^ but the only detergent that SERT was stable in was digitonin,^34^ which is unsuitable for crystallography. Therefore, a different assay where an inhibitor was used to stabilise the transporter was developed. This entailed adding [^125^I]RTI55 to the T-REx-SERT cells followed by detergent solubilisation and then the thermostability assay (heating samples at various temperatures for 30 min). The apparent *T*_m_ was defined as the temperature where 50% of the transporter still bound the radiolabelled inhibitor (Fig. S2). For [^125^I]RTI55-bound SERT, the apparent *T*_m_ was 28 °C, regardless of how it was expressed in HEK293 cells (Fig. S2). Note that a considerable proportion of this thermostability is attributable to the bound inhibitor because the apparent *T*_m_ of *n*-dodecyl-β-d-maltopyranoside (DDM)-solubilised SERT without bound [^125^I]RTI55 could not be measured. [^125^I]RTI55-bound SERT was also sensitive to the concentration of DDM present in the assays (Fig. S2), with the apparent *T*_m_ decreasing as the concentration of detergent increases, similar to that observed for the β_1_-adrenergic receptor.^35^ The most reproducible results with the steepest thermostability curve were obtained with a final concentration of 0.1% DDM; thus, this was used in subsequent assays to determine the thermostability of SERT mutants. The assays were repeated using either [^3^H]imipramine or [^3^H]paroxetine under identical conditions. However, nonspecific binding of both [^3^H]imipramine and [^3^H]paroxetine to DDM micelles was very high (greater than 50% of the total binding measured). In our experience, this would introduce significant experimental variability during the testing of hundreds of mutants, which would result in unreliability and the generation of numerous false positives. We therefore used [^125^I]RTI55 for all subsequent thermostability assays as background binding to DDM micelles was negligible.

### Thermostabilisation of SERT

**Fig. 1:**
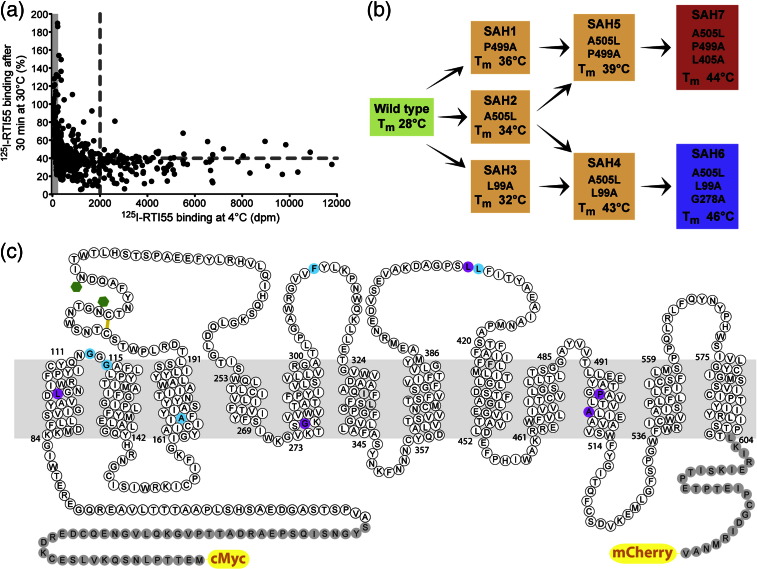
Thermostabilisation of SERT. (a) Comparison of [^125^I]RTI55 bound to 554 detergent-solubilised Ala/Leu mutants either at 4 °C or after heating at 30 °C for 30 min. The data relating to thermostability (the heated samples) have been normalised to the amount of wild-type SERT remaining after heating (40%; horizontal broken line). The expression level of wild-type SERT is indicated by the vertical broken line, and the grey column (0–200 dpm) represents nonspecific binding of [^125^I]RTI55 to the parental T-REx-HEK293 cell line (no SERT). Each data point represents binding to an equivalent of 50,000 cells measured in duplicate (estimated error is ± 20% expressed in dpm). (b) The optimally thermostabilised SAH mutants were engineered by combining the best thermostabilising mutations (as indicated) that resulted in an increased apparent *T*_m_ after solubilisation in 0.1% DDM. (c) Amino acid sequence of SERT showing the positions of the thermostabilising mutations (blue or purple) that consistently gave > 1 °C increase in *T*_m_ compared to wild-type SERT; residues in purple were used to stabilise either SAH6 or SAH7. N-linked glycosylation sites are indicated by the green hexagons, a putative disulfide bond is shown as a yellow line and the N-terminal and C-terminal fusion partners are shown (c-Myc tag and the fluorescent reporter protein mCherry) and indicated.

Systematic alanine-scanning mutagenesis was performed throughout SERT between amino acid residues 49 and 603, with each residue changed to alanine or if the residue was already alanine, then it was changed to leucine. Each SERT-mCherry mutant was sequenced to ensure that only the desired mutation was present. A total of 554 mutants were constructed throughout the transmembrane domains and all loop regions (Fig. 1). The N-terminus and C-terminus were not mutated because these regions are predicted to be disordered and they are therefore unlikely to contribute to the thermostability of SERT. Each mutant was transiently transfected into T-REx-HEK293 cells and expression induced by the addition of tetracycline. Expression was assessed by fluorescence microscopy to ascertain whether the mutant was predominantly either at the plasma membrane or intracellular. The thermostability of each mutant was then determined using a single-temperature thermostability assay and compared to the thermostability of wild-type SERT. Under these assay conditions (see Materials and Methods), the sample was heated at 28 °C for 30 min and approximately 40% of wild-type SERT remained functional. Each batch of mutants tested contained wild-type SERT as a control so that the data between different experiments could be normalised (wild type = 40%). Analysis of the results (Fig. 1) identified 34 mutations that appeared to improve the thermostability of SERT but that did not decrease the levels of expression by more than 70%. Interestingly, there was no linear correlation between the levels of expression and thermostability of the mutants, in contrast to in GPCRs where a weak linear correlation was sometimes observed (*r*^2^ = 0.2).^1^ Of the 34 mutations identified, full thermostability curves showed that 10 mutations improved the thermostability of SERT by at least 1 °C (Table S1). Of these 10 mutations, 7 were in the transmembrane helices and 3 were in the extracellular loops (Fig. 1). Further mutation of these Ala/Leu mutants to other amino acid residues did not significantly improve the thermostability of SERT (Fig. S3).

**Fig. 2:**
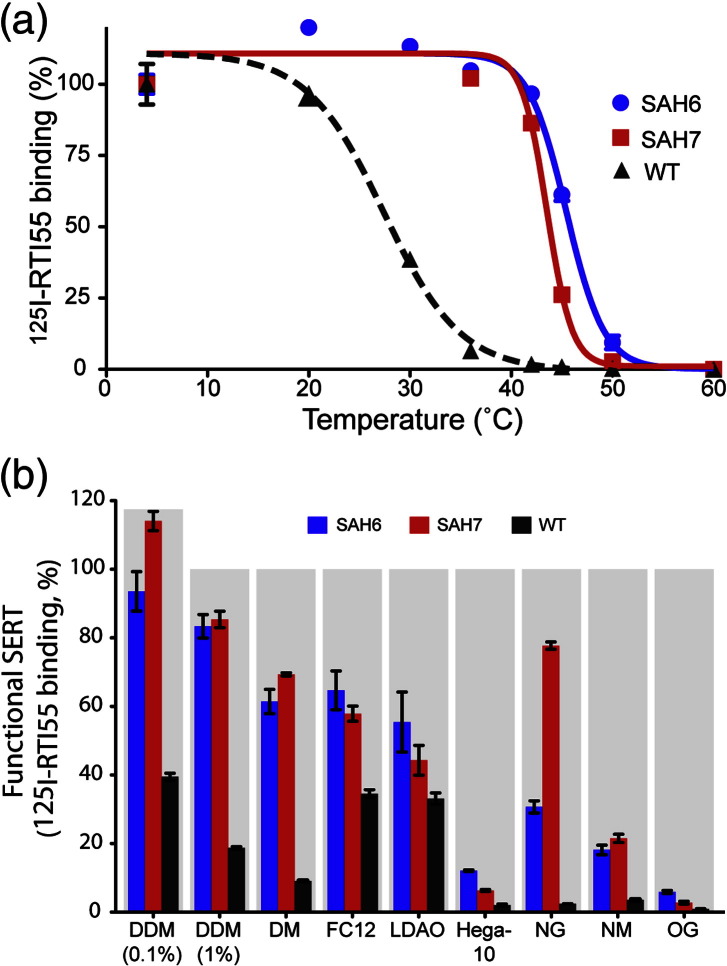
Detergent stability of the thermostabilised mutants SAH6 and SAH7. (a) Thermostability curves for [^125^I]RTI55-bound DDM-solubilised SAH6 (blue circles) and SAH7 (red squares) compared to wild-type SERT (black triangles). The apparent *T*_m_ values determined from the curves are as follows: SAH6, 46 °C; SAH7, 44 °C; wild-type SERT, 28 °C. All the data were collected in a single experiment with measurements performed in duplicate. (b) The stability of [^125^I]RTI55-bound SAH6, SAH7 and wild-type SERT was compared in eight different detergents. [^125^I]RTI55 was added to membranes (final concentration, 1 nM) that were then solubilised for 30 min on ice in the following detergents (aliphatic chain length in parentheses; final detergent concentration, in %): 0.1% DDM (C12), 1% DDM (C12), 0.4% DM (C10), 0.35% FC12 (C12), 0.3% LDAO (C12), 0.6% Hega-10 (C10), 0.5% NG (C9), 0.6% NM (C9), 0.83% OG (C8). The detergent-solubilised samples where then heated at 30 °C for 30 min before determining the amount of SERT remaining in relation to control (incubated on ice). Results are from a single experiment performed in duplicate [± SEM (*s*tandard *e*rror of the *m*ean)] with an equivalent of 50,000 cells per data point.

Combining the thermostabilising mutations in SERT was performed by a rational process previously described for the thermostabilisation of agonist-bound neurotensin receptor and adenosine A_2A_ receptor.^5^ The four best thermostabilising mutations (P499A, A505L, L99A and G113A) were each combined with each other to make a series of double mutants (Table S2). Of these mutants, the most thermostable were A505L + L99A (SAH4) and A505L + P499A (SAH5). These double mutants were then combined with the remaining mutants to make triple mutants (Table S2), with the most thermostable being SAH6 (A505L + L99A + G278A) and SAH7 (A505L + P499A + L405A) with apparent *T*_m_ values of 16 °C and 18 °C higher than that of wild-type SERT (Fig. 2). Further combinations of mutations did not improve significantly the thermostability of these mutants (Table S2); thus, SAH6 and SAH7 were identified as the best candidates for structural studies and were therefore characterised further.

### Characterisation of optimally stabilised mutants SAH6 and SAH7

**Fig. 3:**
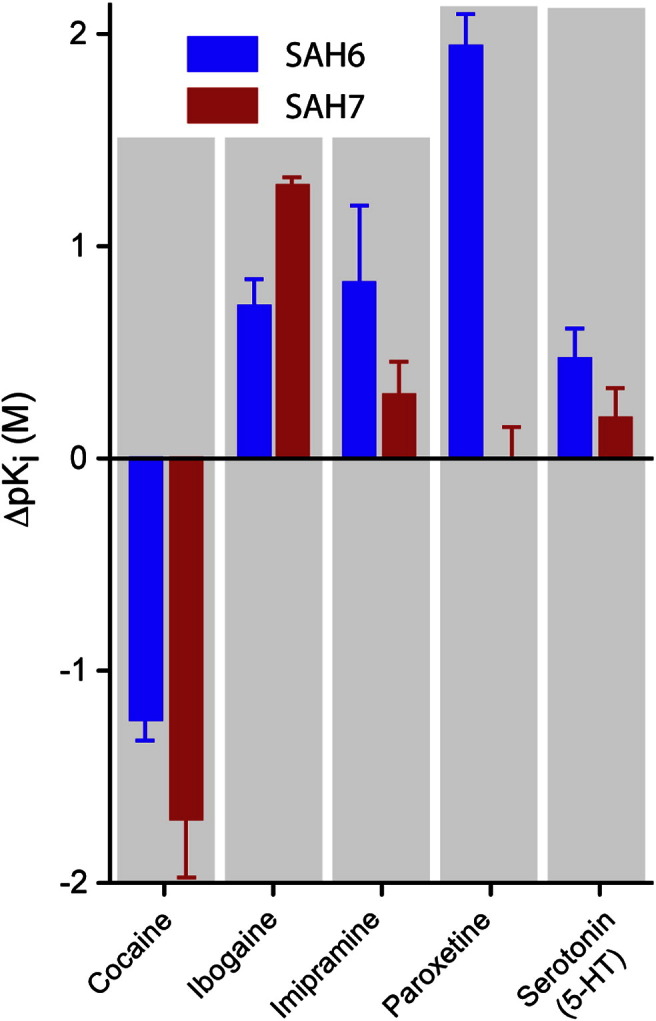
Inhibitor and substrate affinities for SAH6 and SAH7 mutants compared with wild-type SERT. Apparent *K*_i_ values were determined from competition binding curves (Fig. S4) and are plotted as the change in affinities with respect to wild-type SERT (Δp*K*_i_). Competition assays were performed on membranes using a final concentration of 0.2 nM [^125^I]RTI55 and apparent *K*_i_ values determined using the following apparent *K*_D_ values for [^125^I]RTI55 binding (Fig. S4): SAH6, 3.7 ± 0.7 nM; SAH7, 1.2 ± 0.5 nM; wild-type SERT, 3.7 ± 2.2 nM. Error bars are proportional to the standard error of the mean from the original measurements.

**Fig. 4:**
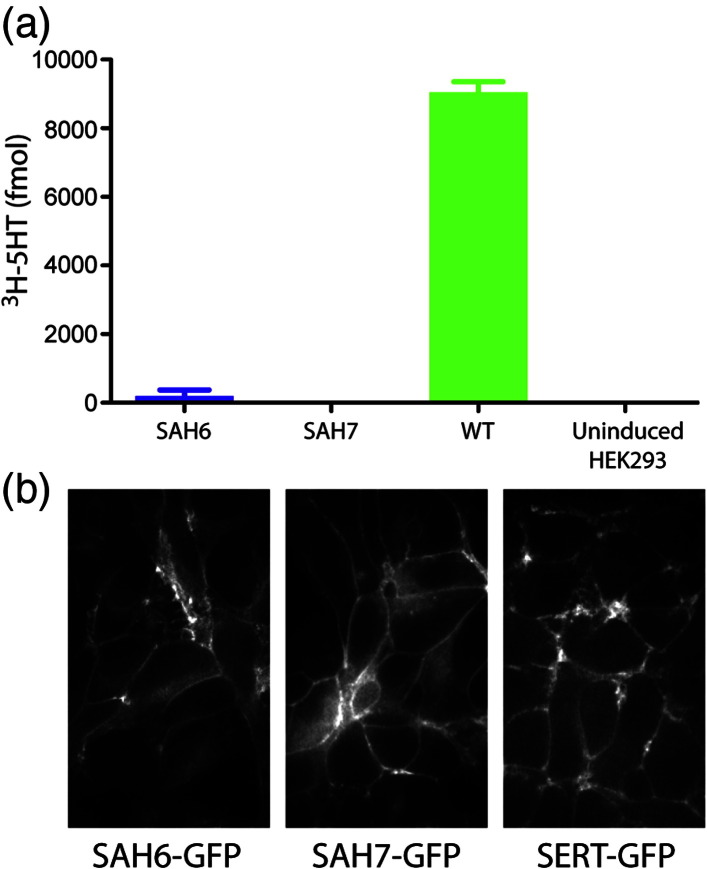
Thermostabilised mutants do not transport 5HT. (a) [^3^H]5HT uptake assays were performed on tetracycline-induced stable T-REx cell lines expressing SAH6-GFP (blue), SAH7-GFP (red) and SERT-GFP (green), with nonspecific uptake determined upon addition of 10 μM cocaine and the results baseline corrected. The results are from a single experiment performed in triplicate [± SEM (*s*tandard *e*rror of the *m*ean)] with approximately 100,000 cells per data point. (b) Confocal microscope images of cells expressing transporter-GFP fusions used in the uptake assays in (a). No fluorescence was detected in parental T-REx-HEK293 cells. SAH6-GFP, SAH7-GFP and SERT-GFP were all capable of binding [^125^I]RTI55 binding with high affinity (Fig. S4).

The most useful characteristic of thermostabilised GPCRs is that they are more stable in short chain detergents that are suitable for crystallography,^1^ and both SAH6 and SAH7 were also more tolerant to short chain detergents than wild-type SERT (Fig. 2). Another defining characteristic of conformationally thermostabilised GPCRs is that the receptors are preferentially in one particular conformation (see Discussion). Therefore, radioligand binding assays were performed on the thermostabilised SERT mutants. The affinity of SAH6 and SAH7 for [^125^I]RTI55 in saturation ligand binding assays was found to be largely unchanged with apparent *K*_D_ values of 3.8 ± 0.1 nM and 1.3 ± 0.1 nM, respectively, compared to 1.6 nM ± 0.1 nM for wild-type SERT (Fig. S4). Competition binding assays (Fig. S4) showed that SAH6 had an apparent affinity for cocaine 17-fold higher than that of wild-type SERT (Fig. 3) whilst there was a small decrease in affinity for ibogaine, imipramine, paroxetine and serotonin (4.5-, 6.7-, 90- and 2.9-fold, respectively). SAH7 showed a similar profile of binding, although the absolute values differed slightly (Fig. S4 and Fig. 3). Although both SAH6 and SAH7 were capable of binding inhibitors and the substrate serotonin, neither mutant was able to efficiently transport [^3^H]5HT into the cell despite the presence of the mutants in the plasma membrane as defined by confocal microscopy (Fig. 4).

## Discussion

Conformational thermostabilisation of GPCRs resulted in a shift in the equilibrium between their two main conformations R and R*, where the R* state can couple to G proteins.^1^ Thus, receptors thermostabilised using antagonists were preferentially in the R state (decreased affinity for agonists and unchanged affinity for antagonists), whilst receptors thermostabilised with agonists were preferentially in an R* state (decreased affinity for antagonists and unchanged/increased affinity for agonists). Transporters are similar to GPCRs because they exist in at least two distinct conformations, with the substrate binding site accessible to either the extracellular environment (outward open) or to the cytoplasm (inward open), with a number of potential intermediate occluded states where the substrate cannot dissociate to either side of the membrane.^36,37^ Indeed, the structures of many bacterial transporters that fit into the above scheme have been determined, and at least in the case of Mhp1^38,39^ and LeuT,^26,40^ different conformations of the same transporter have also been described. With these data in mind, what do the binding studies performed on both SAH6 and SAH7 suggest about their respective conformations?

The affinity of [^125^I]RTI55 for both SAH6 and SAH7 is virtually identical with the wild-type transporter,^28^ which strongly supports the contention that the mutants are folded in a biologically relevant conformation. This is further supported by the cell surface expression of both mutants in stable cell lines expressing either SAH6 or SAH7, as misfolded SERT is retained in the endoplasmic reticulum. Competition assays using both inhibitors and the substrate 5HT provide further evidence on the likely conformation that has been stabilised. Both SAH6 and SAH7 bind cocaine with higher affinity than wild-type SERT. None of the mutations are in the region proposed to be the inhibitor binding site^41,42^; thus, in analogy to what has been previously observed in GPCRs, these data suggest that SAH6 and SAH7 have been stabilised in a “cocaine-bound” conformation. This is perhaps unsurprising given that cocaine represents the core structure of RTI55^32^ and that they therefore bind at a similar site. As cocaine has been proposed to bind preferentially to the outward-open conformation of SERT,^43^ it is likely that both SAH6 and SAH7 are thermostabilised in an outward-open state. The decrease in binding affinity of both imipramine and paroxetine for SAH6 is consistent with this interpretation, as there are likely to be subtle differences between the binding of these inhibitors compared to RTI55, even though they are all proposed to bind to the outward-open state.^44,45^ Comparison of the affinities of inhibitor binding shows that there are subtle differences between SAH6 and SAH7, although why these arise is difficult to ascertain in the absence of a structure.

A characteristic of transporters is obviously their ability to facilitate the vectorial movement of substrates across biological membranes.^36,37^ 5HT transport catalysed by SAH6 and SAH7 was therefore compared with the wild-type transporter in stable cell lines that robustly express the transporters on the cell surface of a tetracycline-inducible HEK293 cell line.^30,46^ Despite robust transport of [^3^H]5HT facilitated by wild-type SERT, no significant transport was observed in cell lines expressing cell-surface-expressed SAH6 or SAH7. This is not due to alterations in the binding site for 5HT because 5HT prevented [^125^I]RTI55 binding in competition assays although the affinity for 5HT was decreased by 1.5- to 2.9-fold. These data are consistent with the theory that both SAH6 and SAH7 are thermostabilised in a specific outward-open conformation. However, at this stage, we cannot categorically confirm this, as there is always a possibility of unexpected dynamics of membrane proteins, which could be a result of the paucity of structural understanding of SERT. Indeed, ibogaine was still capable of inhibiting the binding of [^125^I]RTI55 to both SAH6 and SAH7 (with a change in *K*_i_ of either 4.5-fold or 16.5-fold, respectively) despite the fact that ibogaine has been proposed to bind preferentially to the inward-open conformation.^47^ Whether this is a reflection on the potentially dynamic nature of the thermostabilised mutants or insufficient understanding of the binding of ibogaine to SERT will only be resolved convincingly upon the structure determination of SERT.

**Fig. 5:**
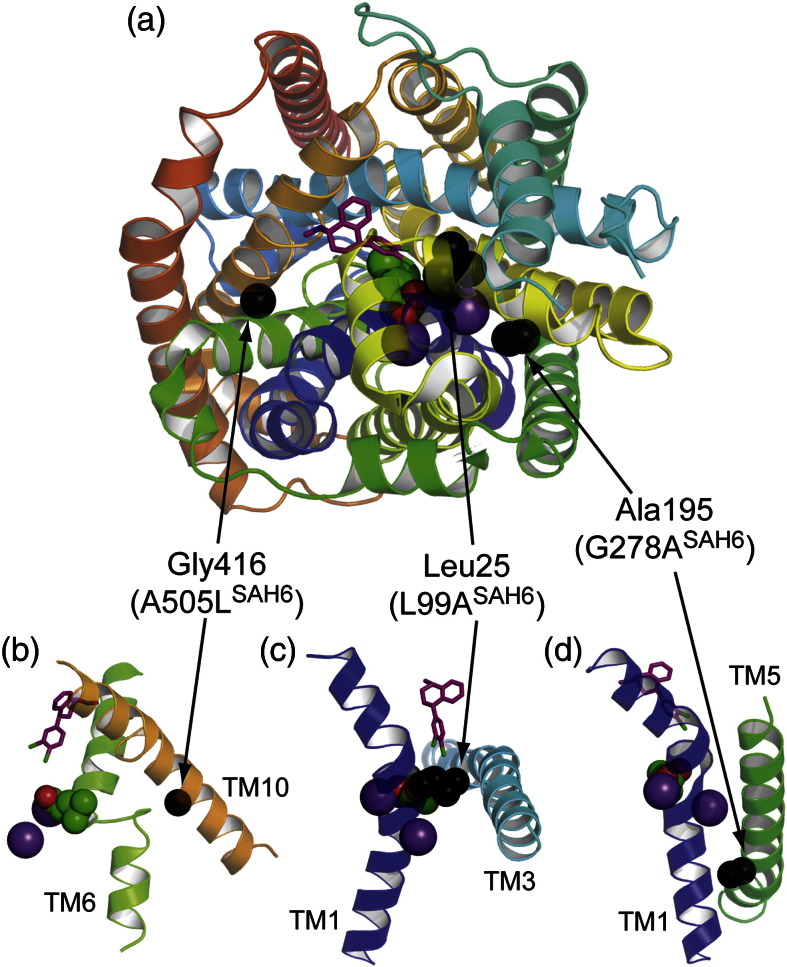
Amino acid residues in LeuT equivalent to the thermostabilising mutations in SAH6. (a) The structure of LeuT (Protein Data Bank code 3GWU) is depicted in rainbow colouration (N-terminus, blue; C-terminus, red) with the side chains in equivalent positions to the thermostabilisation mutations in SAH6 shown as black spheres. The view is from the extracellular surface perpendicular to the membrane plane. (b–d) The mutations are found at helix–helix interfaces and often at the sites of kinks or unwound regions; (b) Gly416^LeuT^ (A505L^SAH6^); (c) Leu25^LeuT^ (L99A^SAH6^); (d) Ala195^LeuT^ (G278A^SAH6^). Additional views of the mutations are in Fig. S6, and for the details of SAH7 mutations, see Fig. S7.

There is a growing body of data suggesting that the structure of SERT is very similar to that of the bacterial transporter LeuT. Indeed, structures of LeuT bound to antidepressant drugs that bind to SERT have been determined^41,42^ and, despite the large difference of binding affinities (millimolar *versus* nanomolar), have led to plausible models for how antidepressant drugs inhibit SERT. We have therefore mapped the thermostabilising mutations we have identified in this study to the structure of LeuT bound to sertraline (Figs. S5–S7 and Fig. 5). It is striking that all the mutations in SAH6 and SAH7 are found at the interfaces between transmembrane α-helices and, more specifically, in either a kinked region or in an α-helix adjacent to a kink. This is analogous to the positions of thermostabilising mutations in GPCRs, although in GPCRs, some mutations were also found in amino acid residues that point into the lipid bilayer.^18,48^ It is tempting to speculate on the role of these amino acid residues in the conformational changes in both SERT and LeuT, but as the structure of SERT has not yet been determined, any proposals would be highly tentative. However, a number of observations that may lead to fruitful avenues of investigation can be made. The conserved nature of the amino acid residues that have been mutated to improve the thermostability of SERT suggests that similar mutations in related transporters such as that for norepinephrine (NET) and dopamine (DAT) may also improve their thermostability, as has been observed when thermostabilising mutations have been transferred between closely related GPCRs.^35^ In addition, the importance of the helix–helix interactions defined by the thermostabilisation mutations may be worth investigating, particularly, in the context of a well-studied transporter such as LeuT, as this may facilitate our understanding of the dynamics of these transporters. Undoubtedly, the structure of a thermostabilised mammalian SERT mutant will be of most benefit to enhance our understanding of the NSS family, and this work is currently in progress.

## Materials and Methods

### Materials

All radiolabelled ligands were purchased from PerkinElmer and detergents were from Anatrace.

### cDNA clones and mutagenesis

The construct c-Myc-SERT-mCherry-BioHis10 was developed from the SERT cDNA in plasmid pCGT137^28^ and inserted into the mammalian cell expression vector pcDNA5/FRT/TO (Invitrogen), which was used for site-directed mutagenesis and expression. The plasmid constructs used for stable expression of SERT-GFP, SAH6-GFP and SAH7-GFP mutants were derived from plasmid pCGT273,^30^ which was modified into SERT-GFP-StrepII-His10 (pJMA111-SAH6 and pJMA111-SAH7). Mutants were generated by PCR using the QuikChange II methodology (Stratagene) using KOD Hot Start polymerase (Novagen). PCR reactions were transformed into XL1-competent cells (Stratagene), and every cDNA clone was fully sequenced to ascertain the presence of only the desired mutation. Mutations were combined by PCR as above, but using multiple primers.

### Transient transfection and generation of stable cell lines

Plasmid DNA for each SERT-mCherry mutant was amplified using a Maxi-prep kit (Qiagen) and transiently transfected (GeneJuice, Novagen) into adherent mammalian T-REx-293 cells (50% confluent) grown in Dulbecco's modified Eagle's media supplemented with 10% tetracycline-free fetal bovine serum and 5 μg/l blasticidin. Expression of mutants was induced by addition of 0.8 μg/ml tetracycline and incubation at 37 °C for 24 h. Stable cell lines expressing SAH6-GFP and SAH7-GFP in T-REx-293 cells were generated by selection with media containing 200 μg/ml zeocin.

### Radiolabelled inhibitor binding assays

Saturation binding curves for membrane-bound SERT were obtained using a range of [^125^I]RTI55 concentrations from 0.13 nM to 160 nM in a 96-well plate format with nonspecific binding being accounted for by incubating identical samples with 1 μM cocaine. The samples were incubated for 2 h at 30 °C and then filtered on 96-well glass fibre plates (Millipore) pre-treated with 200 μl of 0.1% polyethyleneimine. The filters were washed three times with 200 μl ice-cold SERT buffer [100 mM NaCl and 20 mM Tris (pH 7.4)], dried for 1 h at 50 °C prior to liquid scintillation counting. Competition binding assays were performed as above, but a range of concentrations of unlabelled ligand was included and a final concentration of 0.2 nM [^125^I]RTI55 was used.

The thermostability of detergent-solubilised [^125^I]RTI55-bound SERT was determined as previously described for GPCRs.^5,6,8,9^ Briefly, cells containing unpurified SERT were incubated with 1 nM [^125^I]RTI55 for 30 min on ice, which were then solubilised with detergent on ice for 30 min before incubation at varying temperatures for 30 min. The radioligand bound to the membrane protein was separated from free radioligand by centrifugal gel filtration and the radioligand bound to the eluted transporter measured by liquid scintillation counting.

### [^3^H]5HT uptake assays

The [^3^H]5HT uptake assays were performed with slight modifications to the method previously described.^46^ In brief, T-REx-293 cells and T-REx-SERT cells were plated onto poly-l-lysine-coated (1 mg/ml) 24-well plates, grown to 80% confluency, induced by the addition of 0.8 μg/ml tetracycline and grown for 48 h. The growth medium was aspirated and the cells were washed once with TB buffer [10 mM Hepes (pH 7.5), 150 mM NaCl, 2 mM KCl, 1 mM CaCl_2_ and 1 mM MgCl_2_]. The assays were performed at 25 °C using one million cells in 400 μl TB buffer and 2 μM [^3^H]5HT and terminated 3 min after addition of substrate by three washes of ice-cold TB buffer containing 1 μM paroxetine or 10 μM cocaine. [^3^H]5HT was released by rupturing the cells with 2% SDS, which was quantified by liquid scintillation counting. Nonspecific uptake was defined as [^3^H]5HT transport in the presence of 10 μM paroxetine or 10 μM cocaine.
