# Supplementary material for: Thermostabilisation of the Serotonin Transporter in a Cocaine-Bound Conformation
Source: J Mol Biol. 2013 Jun 26;425(12):2198–207. doi: 10.1016/j.jmb.2013.03.025 (PMC3678023; doi:10.1016/j.jmb.2013.03.025)
Supplement: Supplementary file 1 — Supplementary materials [file mmc1.pdf]

a

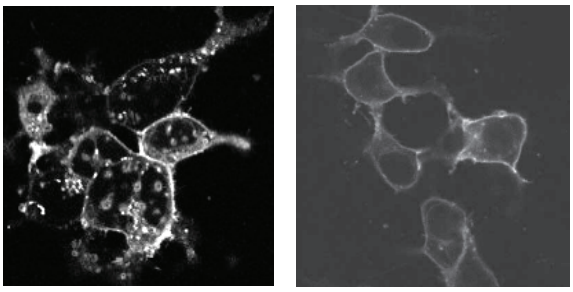

b

Plasmid DNA (μg per 50,000 cells)	0.05	0.1	0.2	0.4	0.8
Plasma membrane expression (%)	85	90	70	30	5

Fig. S1. Optimisation of SERT expression by transient transfection in T-Rex-HEK293 cells.
(a) The amount of tetracycline used to induce cells transiently transfected with SERT-mCherry was tested to give good cell surface expression with minimal intracellular expression, as defined by confocal microscopy. Examples of cells depicted were either induced with 1.2 μg/ml tetracycline (left hand panel) or 0.8 μg/ml tetracycline (right-hand panel). Cells were transfected with 0.1 μg of plasmid DNA per 50,000 cells and were induced for 48 hours.
(b) The DNA:transfection reagent ratio for maximal correctly localised expression of SERT. 50,000 cells were transfected with a range of DNA concentrations. Cells were induced with 0.8 μg/ml tetracycline for 48 hours, examined using a confocal microscope and the percentage of plasma membrane expression of SERT was estimated by eye.

Fig. S2

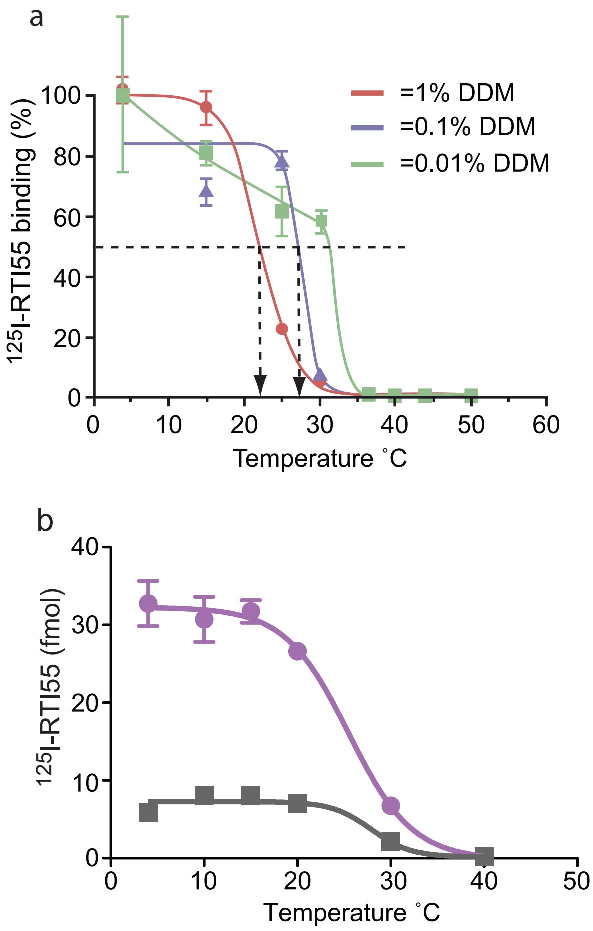

Fig. S2. Development of a thermostability assay for SERT.
(a) The thermostability of ^{125}I-RTI55-bound SERT was determined after solubilisation in three different concentrations of DDM (final concentrations in %): green squares, 0.01%; blue triangles, 0.1%; red circles, 1%. The difference in thermostability is likely a consequence of the degree of delipidation of the transporter that increases as the amount of detergent increases. Each data point (± SEM) was obtained in duplicate from an equivalent of 50,000 cells from a tetracycline-induced stable cell line T-Rex-SERT.
(b) Thermostability assays of SERT-His$_{10}$ expressed in the stable cell line T-Rex-SERT (purple circles) and SERT-mCherry transiently transfected into T-Rex-HEK293 cells (grey squares). Cells were solubilised with 0.1% DDM after the addition of 1 nM ^{125}I-RTI55; the apparent T_m of both samples of SERT was 28°C. All the reactions contained the equivalent of 50,000 cells per data point. The results are from a single experiment performed in duplicate (± SEM).

Fig. S3

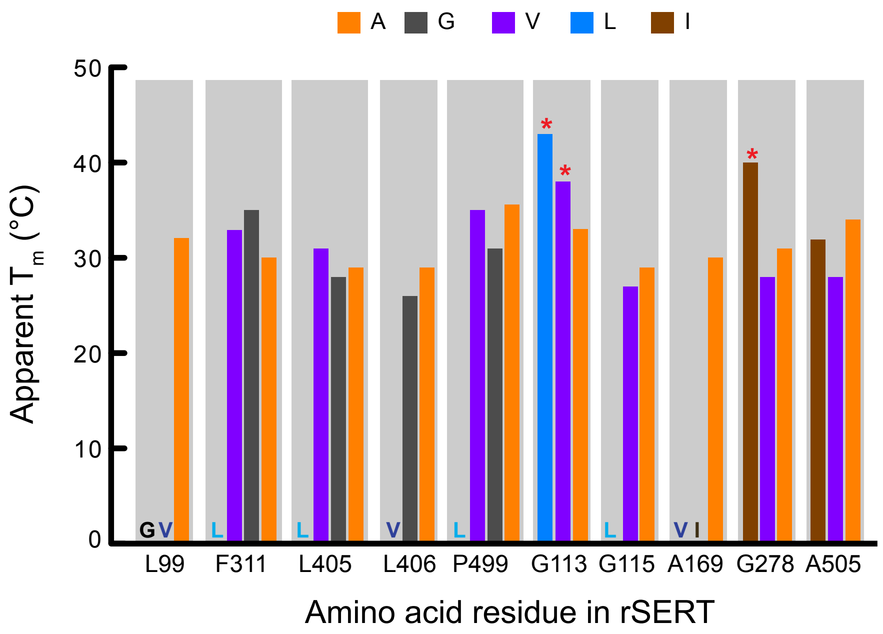

Fig. S3. Alternative amino acid residues for SERT thermostabilisation. Amino acid residues already identified as being thermostabilising in the preliminary Ala/Leu scan were changed either to Ala (orange), Gly (black), Val (purple) Leu (blue) or Ile (brown). Where no functional SERT was detected, the amino acid to which the residue was mutated is given in the single letter code. Bars marked with an asterisk showed improved thermostability over the original mutation, but expression levels were less than 10% of the wild type SERT and therefore these mutations were not used further.

Fig. S4

a

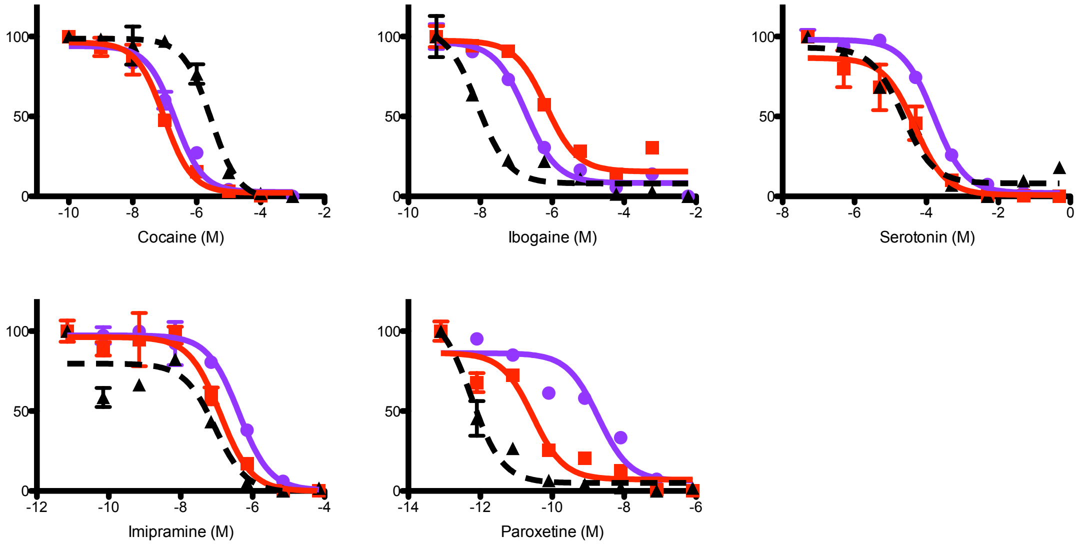

b

K_i	SAH6	SAH7	WT
Cocaine (nM)	90 ± 17	30 ± 21	1560 ± 21
Ibogaine (nM)	90 ± 23	330 ± 19	20 ± 14
Imipramine (nM)	0.2 ± 0.24	0.06 ± 0.02	0.03 ± 0.02
Paroxetine (nM)	0.9 ± 0.01	0.01 ± 0.02	0.01 ± 0.02
Serotonin (μM)	155 ± 37	82 ± 30	53 ± 22

c

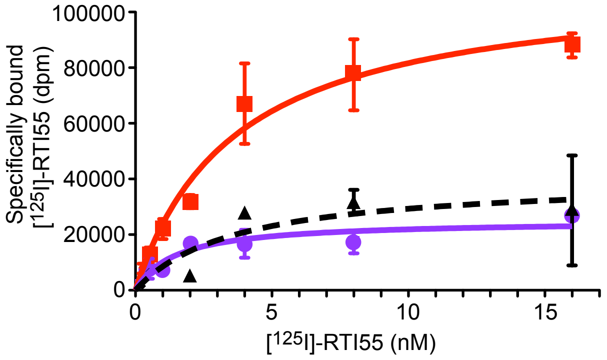

Fig S4 Competition curves for SAH6 and SAH7.
(a) Competition assays were performed on membranes using a final concentration of 0.2 nM ^{125}I-RTI55
(b) Apparent K_i values were determined from the data in (a) using the apparent K_D values for ^{125}I-RTI55 binding determined in (c).
(c) Saturation binding curves of ^{125}I-RTI55 binding to SAH6, SAH7 and SERT. Apparent KD were determined by non-linear regression (GraphPad, Prism): SAH6, 3.7 ± 0.7 nM; SAH7, 1.5 ± 0.6 nM; wild-type SERT, 3.7 ± 2.2 nM.
All results were obtained from two independent experiments performed in duplicate (± SEM) .
For all graphs: wild type SERT, black triangles; SAH6, blue circles; SAH7, red squares.

```
SERT    1 METTPLNSQKVLSECKDREDCQENGVLQKGVPTTADRAEPSQISNGYSAVPSTSAGDEAS  60
LeuT    1 ------------------------------------------------------------   0

SERT   61 HSIPAATTTLVAEIRQGERETWGKKMDFLLSVIGYAVDLGNIWRFPYICYQNGGGAFLLP 120
LeuT    1 --------------MEVKREHWATRLGLILAMAGNAVGLGNFLRFPVQAAENGGGAFMIP  46
                         .  ** *  ..  .*.. * ** ***  ***  . .******..*

SERT  121 YTIMAIFGGIPLFYMELALGQYHRNGCISIWRKI------CPIFKGIGYAICIIAFYIAS 174
LeuT   47 YIIAFLLVGIPLMWIEWAMGRYGGAQGHGTTPAIFYLLWRNRFAKILGVFGLWIPLVVAI 106
          * *  .  ****  .* *.*.*           *            .*     *   .*

SERT  175 YYNTIIAWALYYLISSLTDRLPWTSCTNSWNTGNCTNYFAQDNITWTLHSTSPAEEFYLR 234
LeuT  107 YYVYIESWTLGFAIKFLVGLVPEP----------------PPNATDPDSILRPFKEFLYS 150
          **  * .*.* . *  *   .*                    * *       *  **

SERT  235 HVLQIHQSKGLQDLGTISWQLTLCIVLIFTVIYFS-IWKGVKTSGKVVWVTATFPYIVLS 293
LeuT  151 YIGVPKGDEPILKPSLFAYIVFLITMFINVSILIRGISKGIERFAKIAMPTLFILAVFLV 210
           .   .    .      .  . *  . *   *    * **.    *.   *     . *

SERT  294 VLLVRGATLPG-AWRGVVFYLKPNWQKLLETGVWVDAAAQIFFSLGPGFGVLLAFASYNK 352
LeuT  211 IRVFLLETPNGTAADGLNFLWTPDFEKLKDPGVWIAAVGQIFFTLSLGFGAIITYASYVR 270
          . .    *  * *  *. *   *  .** . ***. *  ****.*  *** ....*** .

SERT  353 FNNNCYQDALVTSVVNCMTSFVSGFVIFTVLGYMAEMRNEDVSEVAKDAGPSLLFITYAE 412
LeuT  271 KDQDIVLSGLTAATLNEKAEVILGGSISIPAAVAFFG-VANAVAIAKAGAFNLGFITLPA 329
            .      * .. .*  .  . *  *  .              .**     * ***

SERT  413 AIANMPASTFFAIIFFLMLITLGLDSTFAGLEGVITAVLDEFPHIWAKRREWFVLIVVIT 472
LeuT  330 IFSQTAGGTFLGFLWFFLLFFAGLTSSIAIMQPMIAFLEDELKLSRKHAVLWTAAIVFFS 389
            ..  . **   . * .*   ** *. * .. .*. . **   .  .   *   **  .

SERT  473 CVLGSLLTLTSGGAYVVTLLEEYATGPAVLTVALIEAVAVSWFYGITQFCSDVKEMLGFS 532
LeuT  390 AHLVMFLN------KSLDEMDFWAGTIGVVFFGLTELIIFFWIFGADKAWEEINRGGIIK 443
          . *   *. .    . .  ..  * .  *.   * * .   * .*  .   ..

SERT  533 PGWFWRICWVAISPLFLLFIICSFLMSPPQLRLFQYNYPHWSIVLGYCIGMSSVICIPTY 592
LeuT  444 VPRIYYYVMRYITPAFLAVLLVVWAREYIPKIMEETHWTVWITRF-YIIGLFLFLTFLVF 502
                     *.* **  ..           . . .   *     * **.   ..   .

SERT  593 IIYR--LISTPGTLKERIIKSITPETPTEIPCGDIRMNAV 630
LeuT  503 LAERRRNHESAGTLVPR-----------------------  515
          .  *      .***  *
```

Fig. S5. Amino acid sequence alignment between *Rattus norvegicus* SERT and *Aquifex aeolicus* LeuT (19% identity). Identical residues (*) and similar residues (.) are indicated below the aligned amino acid sequences. The transmembrane α-helices in LeuT (as defined by the structure) are shown as red bars, with unwound regions as a red line, and other α-helices are shown as blue bars. The positions of the 11 thermostable mutations are shown either in purple (present in SAH6 and/or SAH7) or pale blue (other thermostabilising mutations).

Fig. S6

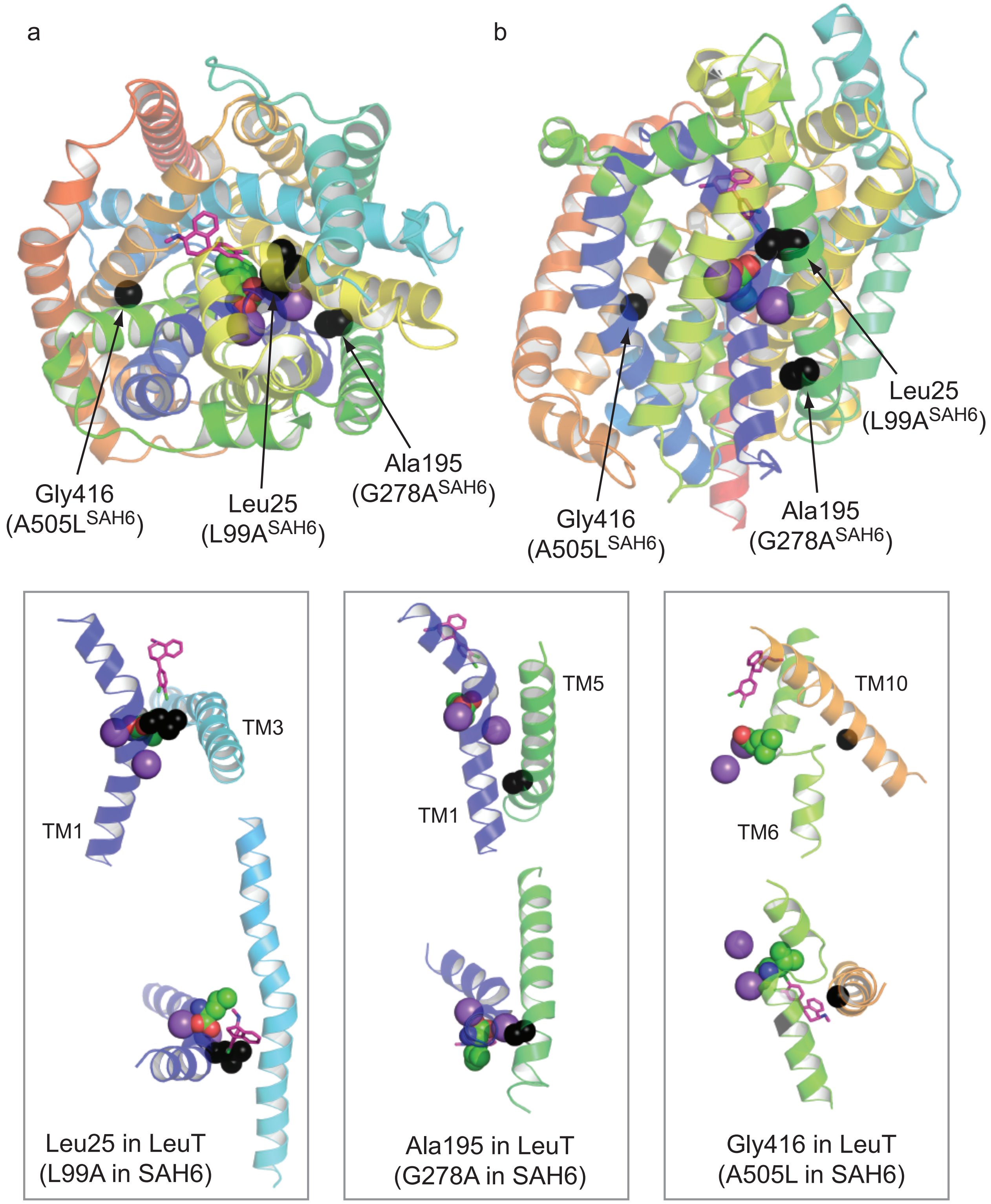

Fig. S6. Equivalent positions in LeuT of the thermostabilising mutations in SAH6. A cartoon of LeuT (PDB code 3GWU) is shown in rainbow colouration (N terminus blue, C-terminus red) with bound leucine (spheres: C, green; O, red; N, blue), Na^+ ions (purple spheres) and sertraline (sticks: C, magenta; N, blue; fluorine, green). Black spheres indicate amino acid side chains in LeuT that are equivalent to the thermostabilising mutations found in SAH6 (in parentheses): Leu25 (L99A); Ala195 (G278A); Gly416 (A505L). LeuT is shown either viewed from the extracellular surface of the membrane or parallel to the membrane plane. Portions of the structures have been highlighted (boxes) to depict the position of the residues in relation to helix-helix interfaces.

Fig. S7

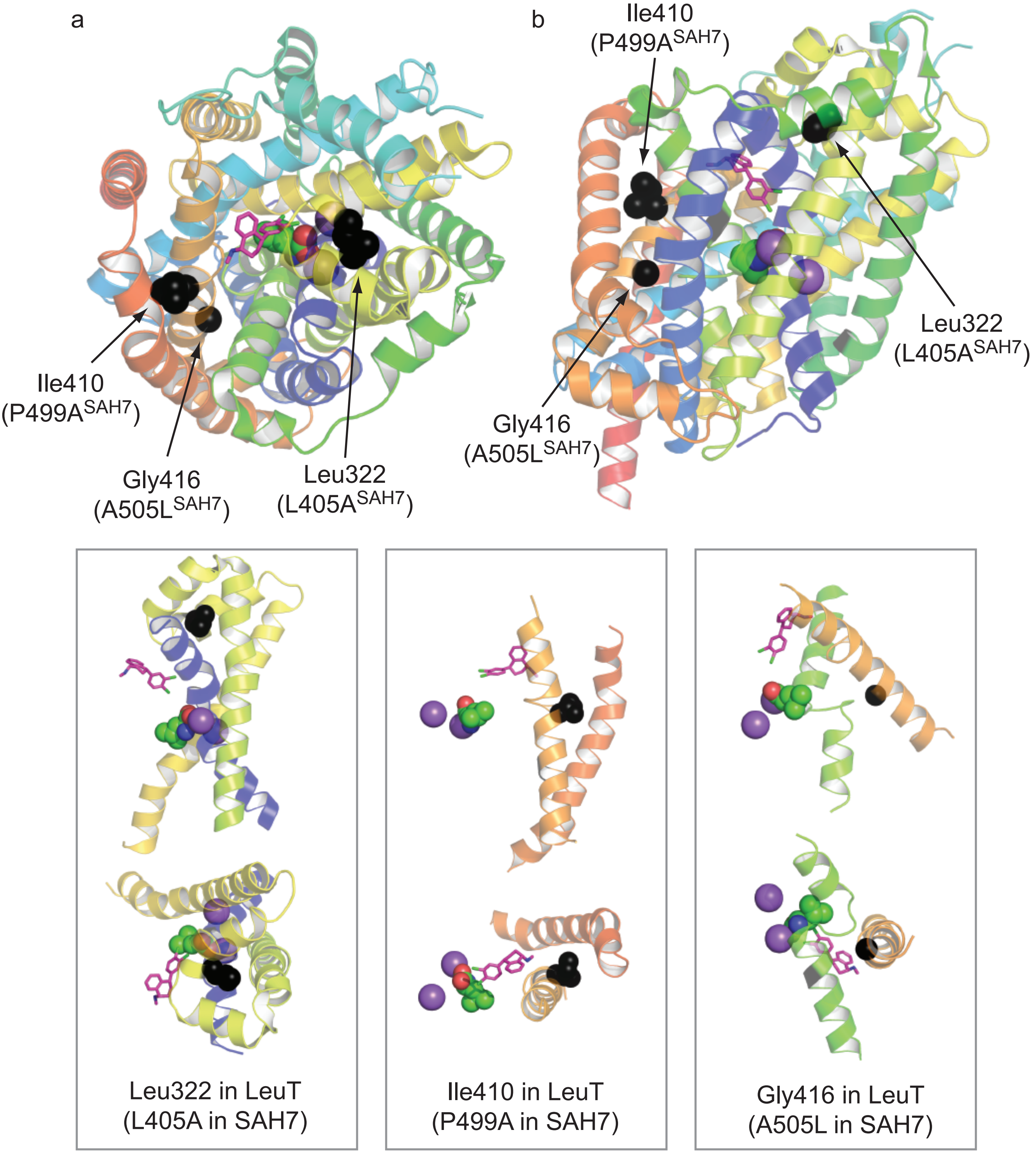

Fig. S7. Equivalent positions in LeuT of the thermostabilising mutations in SAH7.
A cartoon of LeuT (PDB code 3GWU) is shown in rainbow colouration (N terminus blue, C-terminus red) with bound leucine (spheres: C, green; O, red; N, blue), Na^+ ions (purple spheres) and sertraline (sticks: C, magenta; N, blue; fluorine, green). Black spheres indicate amino acid side chains in LeuT that are equivalent to the thermostabilising mutations found in SAH7 (in parentheses): Leu322 (L405A); Ile410 (P499A); Gly416 (A505L). LeuT is shown either viewed from the extracellular surface of the membrane (a) or parallel to the membrane plane (b). Portions of the structures have been highlighted (boxes) to depict the position of the residues in relation to helix-helix interfaces.

Mutation	Expression (%) SERT = 100%	Cell surface expression	Apparent T_m (˚C)	ΔT_m (˚C)
**P499A**	65	**	35	7
**A505L**	175	***	34	6
**G113A**	42	**	33	5
**L99A**	131	**	32	4
**G278A**	39	*	31	3
**A169L**	31	**	30	2
**F311A**	492	**	30	2
**G115A**	327	***	29	1
**L405A**	543	***	29	1
**L406A**	87	*	29	1

Table. S1. Thermostability and expression data for the most thermostable Ala/Leu mutants. The top 34 SERT mutants as estimated from the single point thermostability assay were re-tested using a 6-point thermostability curve to determine an accurate apparent T_m and expressed as an improvement in T_m (ΔT_m) assuming wild-type SERT had an apparent T_m of 28˚C. Each mutant was also assessed for cell surface expression as determined by estimation by eye of fluorescence throughout the cell upon confocal microscopy: *, low expression; ** as wild-type SERT, ***, higher expression than wild-type SERT.

	SERT mutation	Apparent T_m in 0.1% DDM
Wild type SERT	none	28°C
Double mutants	A505L+P499A (SAH5)	39°C
	A505L+L99A (SAH4)	43°C
	P499A+L99A	35°C
	P499A+F311A	-
	A505L+F311A	32°C
	L99A+F311A	27°C
Triple mutants	A505L+P499A+L405A (SAH7)	44°C
	A505L+P499A+A169L	33°C
	A505L+P499A+F311A	44°C
	A505L+P499A+L99A	-
	A505L+L99A+G278A (SAH6)	46°C
	A505L+L99A+F311A	43°C
	A505L+L99A+L405A	43°C
	A505L+L99A+L406A	44°C
	A505L+L99A+N101A	40°C
	A505L+L99A+G115A	44°C
	A505L+F311A+L99A	40°C
Quadruple mutants	A505L+P499A+L405A+F311A	44°C
	A505L+P499A+L405A+G115A	45°C
	A505L+L99A+G278A+F311A	44°C
	A505L+L99A+G278A+G115A	44°C
	A505L+L99A+G278A+L405A	44°C

Table S2. Thermostability of double and triple mutants. Combinations of SERT mutants tested for the thermostabilisation of ^{125}I-RTI55-bound detergent-solubilised SERT (apparent T_m ±1°C).
